# End-Functionalized Poly(*N*-isopropylacrylamide) with d-Glucosamine through Different Initiator from C-1 and C-2 Positions via Atom Transfer Radical Polymerization

**DOI:** 10.3390/ma9110913

**Published:** 2016-11-10

**Authors:** Guihua Cui, Zhengguo Gao, Nannan Qiu, Toshifumi Satoh, Toyoji Kakuchi, Qian Duan

**Affiliations:** 1 Department of Chemistry, Jilin Medical College, Jilin 132013, China; cuiyuhan1981_0@sohu.com; 2 Department of Materials Science and Engineering, Changchun University of Science and Technology, Changchun 130022, China; jlmukjc@126.com; 3 Chemical and Engineering College, Yantai University, Yantai 264005, China; zggao_80@163.com; 4 Division of Biotechnology and Macromolecular Chemistry, Graduate School of Engineering, Hokkaido University, Sapporo 060-8628, Japan; Satoh@polymer.ac.jp (T.S.); Kakuchi@polymer.ac.jp (T.K.)

**Keywords:** poly(*N*-isopropylacrylamide) (PNIPAM), d-glucosamine (GA), atom transfer radical polymerization (ATRP), lower critical solution temperature (LCST)

## Abstract

Regioselective modification of d-glucosamine (2-amino-2-deoxy-d-glucopyranose, GA) through C-1 and C-2 positions to synthesized thermo-responsive D-Glucosamine-poly(*N*-iso-propylacrylamide) (PNIPAM) via atom transfer radical polymerization (ATRP) was investigated for the first time. Two different schemes of the synthesis for GA derivatives (GA-PNIPAM (i) and (ii)) with well-defined structures using 3,4,6-tri-*o*-acetyl-2-deoxy-2-phthalimido-*β*-d-glucopyranose and 1,3,4,6-tetra-*o*-acetyl-2-amino-2-deoxy-*β*-d-glucopyranose intermediates were examined. The GA-PNIPAM (ii) had an amino at C-2 position, while there was a hydroxyl in GA-PNIPAM (i) at this position. Both the resulting oligomers (i) and (ii) had a narrow dispersity, and no significant cytotoxic response of copolymers (i) and (ii) was observed in the cell line over the concentration range from 0.1 μg/mL to 1000 μg/mL at any of the exposure times. In addition, it was discovered that GA-PNIPAM (i) and (ii) inhibited the proliferation of Human Hepatocellular Carcinoma Cells HepG2 as the concentration and the time changed, and the inhibitory activity of polymer (ii) was higher than that of he (i). The results suggest that the GA-PNIPAM polymers show excellent biocompatibility in vitro.

## 1. Introduction

The compound 2-amino-2-deoxy-d-glucopyranose (d-glucosamine, GA) is abundant among the natural polysaccharides and glycoconjugates [1,2] and is a carbohydrate component of many cellular glycoproteins, glycolipids and glycosaminoglycans; consequently, it plays an important role on cell surfaces [3,4,5]. GA is a potent inhibitor for several types of tumor cells and acts without influencing normal cells [6,7,8,9]. The difference in reactivity between amino and hydroxyl groups in GA allows regioselectivity to synthesize derivatization with new features and functions [10,11,12]. These synthetic GA derivatives have demonstrated potent biological activity and are used in medicinal research [13,14,15,16,17]. Especially amino oligosaccharides and glycoconjugates are important for developing new biologically active materials with anti-cancer activity. 

To explore the applications of GA derivatives further, GA could be polymerized to form a polymer with a stimuli-responsive architecture: this polymer might lead to derivatives that swell as a response to changes in environmental conditions such as temperature, pH, ionic strength, etc. [18]. Poly (N-isopropylacrylamide) (PNIPAM) is a well-known thermo-responsive polymer that changes its appearance in water from a clear solution to a turbid suspension at a relatively low critical solution temperature (LCST) of 32 °C, a temperature near that of the human body [19,20]. PNIPAM and its related polymers are widely applied for drug delivery and cell therapy.

A series of studies on the glucosamine-PNIPAM polymers have been reported for drug release or self-regulated insulin delivery systems. Typical examples are gels or microgels composed of poly-(acryloyl glucosamine) (PAGA) and PNIPAM [18,21], which can swell and release the drug in response to external environmental changes. It is known that most solid tumors have an acidic extracellular pH (6.85–6.95); obviously, the amino group of the GA played an important role in tumor suppression. However, the above polymers were N-acetyl derivatives and were synthesized by using the modification of GA through the C-2 amino group [22,23,24]. In addition, these glucosamine-carrying temperature- and pH-sensitive polymers were also prepared by free radical precipitation polymerization, and the molecular weight distributions of the polymers were broad and they were unable to control the distribution of functional groups. So far, only a few reports have been related to the C1 hydroxyl group derivatives of GA [25,26,27], and no reports have been related to prepare an initiator using the C1 hydroxyl group of GA for ATRP and synthesize end-glycosylational polymers. In this work, we take the advantages of ATRP to synthesize GA-terminated PNIPAM, and demonstrate that the thermo-responsive polymers can be used to inhibit cancer cell proliferation.

## 2. Materials and Methods

### 2.1. Materials and Instrumentation

*N*-isopropylacrylamide (Aldrich, Cleveland, OH, USA, 98%) was recrystallized twice from a hexane/benzene mixture (3/2, *v*/*v*). Tris(2-(dimethylamino)ethyl)amine (Me_6_TREN) was synthesized from tris(2-amino-ethylamine) (TREN, Aldrich, 99%) according to the literature [28]. CuBr (Aldrich, 99%) was washed successively with acetic acid and ether and then dried and stored under nitrogen. 2-Bromo-2-methylpropionic acid (Acros, Geel, Belgium, 98%), 2-Bromopropionyl bromide (Aldrich, 97%), d(+)-glucosamine hydrochloride (Aladdin, Shanghai, China, 99%) and chemical reagents were obtained commercially and were used as received unless otherwise stated. The 3-[4,5-dimethylthiazol-2-yl]-2,5-diphenyltetrazolium (MTT) was purchased from Acros. Human Hepatocellular Carcinoma Cells HepG2 in culture and NCTC clone 929 cells (L-929) were purchased from the Type Culture Collection of the Chinese Academy of Sciences (Shanghai, China).

The ^1^H nuclear magnetic resonance (NMR) spectra of monomers and polymers in CDCl_3_ were obtained on a Varian Unity 400 NMR spectrometer. Molecular weights (*M*_n_) and polydispersity (*M_w_*/*M*_n_) were measured using a gel permeation chromatograph (GPC), a Waters 510 pump and a Model 410 differential refractometer at 25 °C. THF was used as the mobile phase at a flow rate of 1.0 mL·min^−1^. The LCSTs of the polymer solutions were determined by absorbance at 500 nm, using a Shimadzu-2600 UV-Vis spectrophotometer with a heating rate of 0.1 °C·min^−1^. The LCST was defined as the temperatures corresponding to 10% decrease of transmittance. Polymer concentration was 1 mg/mL. Cell viability was evaluated by MTT, the optical density (OD) was measured at 490 nm with a microplate reader (Bio-Rad, Hercules, CA, USA). Cell viability was determined as a percentage of the negative control (untreated cells).

This section may be divided by subheadings. It should provide a concise and precise description of the experimental results, their interpretation as well as the experimental conclusions that can be drawn.

### 2.2. General Procedure for Synthesis of Polymers

**Scheme 1:**
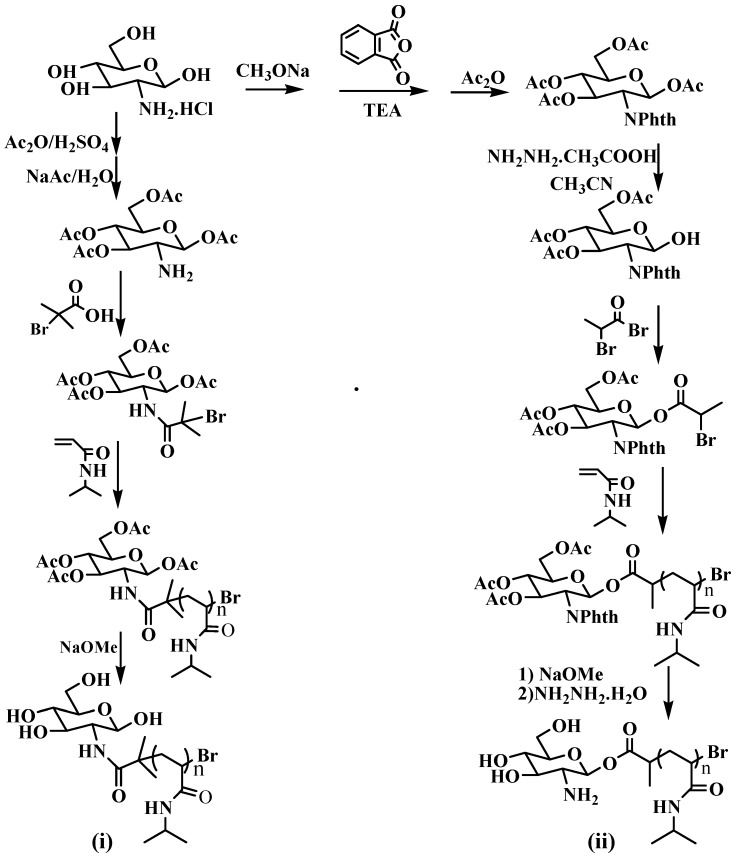
GA-PNIPAM (**i**) and (**ii**) synthesis by ATRP.

GA-PNIPAM (i) and (ii) were synthesized as follows (Scheme 1). A mixture of CuBr and Me_6_TREN (1:1, *v*/*v*) in DMF/H_2_O (95/5, *v*/*v*) was placed on one side of an H-shaped ampoule glass and stirred. A mixture of NIPAM and an initiator in DMF was placed on the other side of the ampoule. Nitrogen was bubbled through both mixtures for 5 min to remove any oxygen. Three freeze-pump-thaw cycles were performed to degas the solutions. The ampoule was placed at room temperature for several hours. The reaction mixture was then diluted with DMF and purified using a neutral Al_2_O_3_ column. After evaporation of the solvent, the crude product was purified using column chromatography (silica gel) to obtain polymers as a white power. Then deacetylated for protected hydroxy in the sodium methylate and methanol solution to obtain GA-PNIPAM (i); removed the phthalimide group from the protected amino group to obtain GA-PNIPAM (ii).

### 2.3. Biocompatibility Study

Cell viability was investigated using NCTC clone 929 ells (L-929) and Human Hepatocellular Carcinoma Cells HepG2 in culture. After incubation for 24 h in 96-well plates (8 × 10^4^ cells/mL per well) using Dulbecco’s modified Eagles medium (DMEM) in an incubator (37 °C, 5% CO_2_), the culture medium was mixed with 200 μL of DMEM containing a sample of PNIPAM homopolymer, GA, GA-PNIPAM (i) and (ii) with a range of sample concentrations from 0.1 to 1000 μg/mL (according to the literature [18]): the mixture was further incubated for 48 h. Each sample was tested in six replicates per plate. Then, 20 µL of MTT solutions was added to the mixture in each well, which was incubated for a additional 4 h. Next, 200 μL of DMSO was added, and the mixtures were shaken at room temperature. Six replicate wells were used for the control and test concentrations for each microplate. In addition, HepG2 cell suspension concentrations of 1 × 10^5^ cells/mL, 6 × 10^4^ cells/mL, and 4 × 10^4^ cells/mL were used the longer duration exposure experiments (24 h, 48 h, 72 h and 96 h exposures, respectively) at each time-point of samples. The cell viability (%) was calculated according to the following Equation (1):

Cell viability(%) = (A _sample_/A _control_ ) × 100% (1)

where A _sample_ was the absorbance of the cells incubated in DMEM and mixture and A _control_ was the absorbance of the cells incubated in DMEM.

## 3. Results and Discussion

### 3.1. Analysis of Polymer Architecture

To the best of our knowledge, there were no reports on the ATRP of NIPAM with glucosamine derivatives as the initiator. The GA initiator and GA-PNIPAM were characterized by ^1^HNMR, GPC, FT-IR. Using an NIPAM/GA initiator/CuBr/Me_6_TREN feed ratio of 50:1:1:1, we could obtain different conversion percents and products with different $M_{n}$ values. The data in Table 1 show that the polymers had a narrow molecular weight distribution, the $M_{w}/M_{n}$ of the GA-PNIPAM (i) and (ii) remained narrow with values in the range of 1.14 to 1.18 and 1.09 to 1.17, respectively. Compared with the GA-PNIPAM (i) system, the conversion rate and molecular weight of the C-1 initiator of GA were low at the initial reaction; this may be caused by the stereospecific blockade of the phthalimide. As the reaction proceeded, the polymer chain extended in an equatorial bond position of the chair conformation of *β*-d-glucosamine to overcome the resistance.

**Figure 1:**
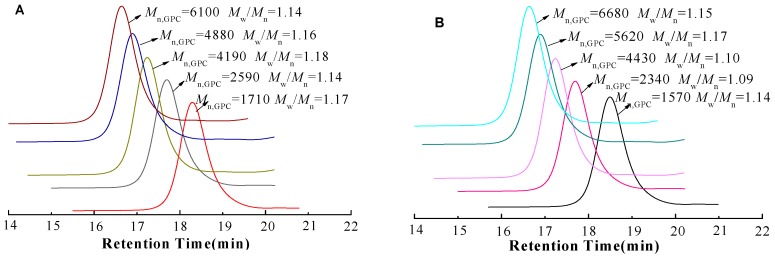
GPC traces of (**A**) GA-PNIPAM (i); (**B**)GA-PNIPAM (ii) obtained through ATRP.

After a series of purifications, GPC traces of polymers (shown in Figure 1) were relatively symmetric and showed no tailing at either side, suggesting the absence of any small molecular residues in the final product, such as the initiator, monomer or other byproducts.

**Figure 2:**
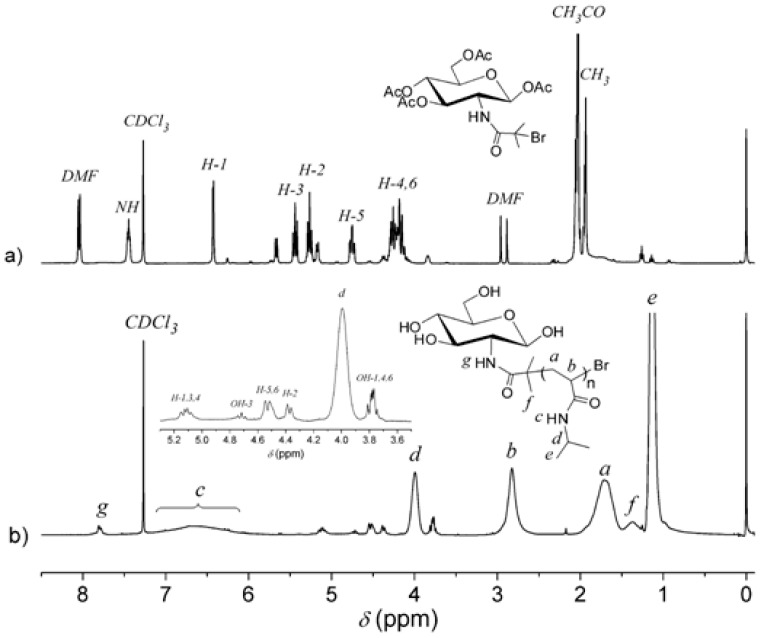
^1^HNMR spectra of (**a**) the GA initiator (i) and (**b**) GA-PNIPAM (i), both in CDCl_3_.

To prepare a well-defined polymer, synthesizing the two pure precursors as an initiator is necessary. Figure 2 shows the ^1^HNMR spectra of the initiator and the product for (i). In Figure 2a, the peaks located at 8.05 ppm and in the range of 5.52 to 4.06 ppm were ascribed to the protons adjacent to GA, whereas the signals at 1.93 ppm in the range of 2.15 to 1.98 ppm corresponded to the methyl and acetyl groups, respectively; these peaks revealed that bromide was successfully introduced into the GA, protected by acetyl groups. The signals at 6.55, 4.0, 2.93, 1.60 and 1.02 ppm in Figure 2b were attributed to the protons of the repeating units of NIPAM, and the signal at 8.50 ppm was the characteristic signal of protons adjacent to nitrogen atoms.

**Figure 3:**
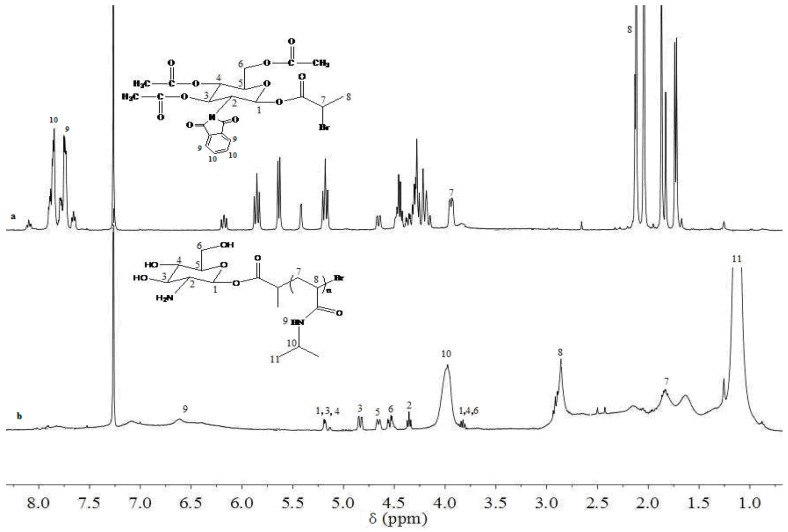
^1^HNMR spectra of (**a**) GA initiator (ii) and (**b**) GA-PNIPAM (ii), both in CDCl_3_.

Figure 3 showed the ^1^HNMR spectra of the initiator and the product for (ii). In Figure 3a, the peaks located at 8.10 ppm in the range of 6.12 to 4.05 ppm were ascribed to the protons adjacent to GA, those at 7.90 to 7.74 ppm were due to the phthalimide, and the signals at 1.93 ppm in the range of 2.15 to 1.98 ppm corresponded to the methyl and acetyl groups, respectively; these peaks revealed that the GA initiator (ii) was synthesized successfully by 1-hydroxy-3,4,6-tri-*O*-acetyl-2-amino-phtalic acid-*β*-d-glucose and 2-Bromopropionyl bromide. The signals at 6.55, 4.0, 2.93, 1.60 and 1.02 ppm in Figure 3b were attributed to the protons of the repeating units of NIPAM, and the signal at 8.50 ppm was the characteristic signal of protons adjacent to nitrogen atoms. We can clearly observe the peaks for each hydroxyl of the GA. Since the hydrogen atoms of the amino acid are very active, we cannot observe the amino signal peaks in deuterated reagents, and the peaks at 1.7 ppm to 1.4 ppm may be the superposition of the amino acid with water or NIPAM repeat units. However, we measured the aqueous solution of the polymer which was alkaline in this experiment.

**Figure 4:**
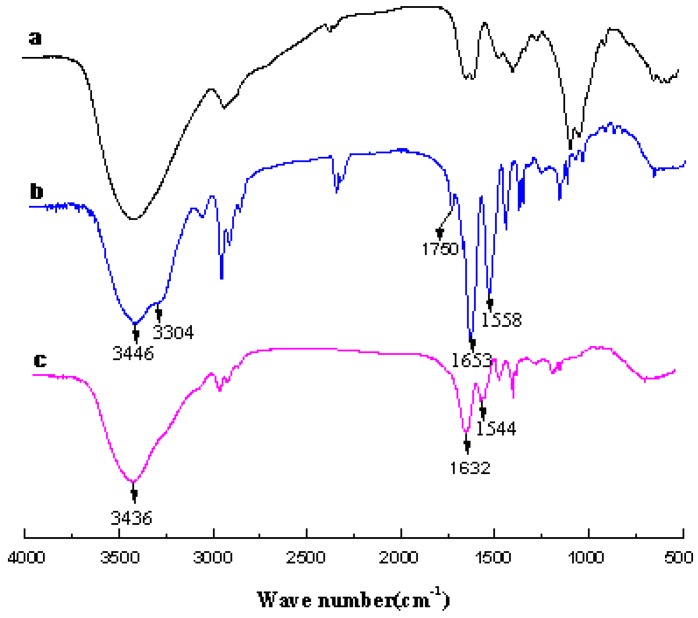
FT-IR spectra of (**a**) GA; (**b**) GA-PNIPAM (ii); (**c**) GA-PNIPAM (i).

The structure of GA-PNIPAM (i) and (ii) were also characterized by the FT-IR spectrum (as shown in Figure 4). The absorption peaks characteristic of PNIPAM can be clearly observed, as evidenced by the presence of the band at 3446 cm^−1^ in Figure 4b, which was assigned to the stretching vibration (*ν*_N–H_) of the acylamino group. The band at 1653 cm^−1^ was ascribed to amide I (mainly the carbonyl stretching vibration (*ν*_C=O_)) and the band at 1558 cm^−1^ was ascribed to amide II (mainly the N–H bending vibration (*δ*_N–H_)), and in Figure 4c, these peaks appeared at 3436 cm^−1^, 1632 cm^−1^ and 1544 cm^−1^, respectively. As seen in Figure 4b, the peak at 1750 cm^−1^ which corresponds to ester group absorption disappeared in Figure 4c, and at 3304 cm^−1^ in the Figure 4b the typical bands of the amino groups of GA-PNIPAM (ii) are visible. Thus, it was suggested that the well-defined GA-PNIPAM (i) and (ii) were successfully prepared through ATRP using a different initiator.

### 3.2. Thermo-Responsivity Property of GA-PNIPAM (i) and (ii)

**Figure 5:**
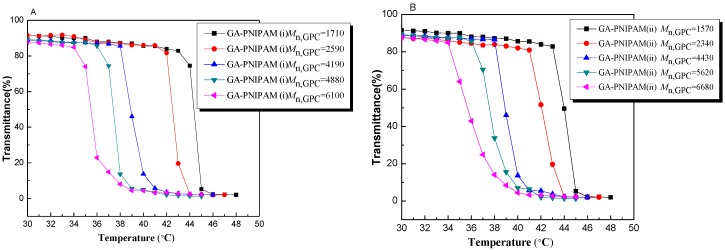
Temperature dependence of optical transmittance at wavelength of 500 nm obtained for aqueous solutions of (**A**) GA-PNIPAM (i) and (**B**) GA-PNIPAM (ii) (1 mg/mL).

Aqueous PNIPAM solutions show a LCST, which has been attributed to the association of polymer molecules through intermolecular hydrogen bonds and non-polar bonds creating large aggregates. When PNIPAM contains different amounts of hydrophilic glucosamine moieties, LCST changes. The LCSTs of the copolymer solutions were determined by turbidimetry. The results are shown in Table 2 and Figure 5. From the GA-PNIPAM (i) polymer P1–P5, the highest LCST was observed at 43.6 °C for 19.19% of GA (P1), which was shifted to 8.9 °C for 5.37% of GA (P5). LCST declined to 9.2 °C of the GA-PNIPAM (ii) polymer from P1 to P5, and all the results indicated that the hydrophilic GA could improve the LCST of the polymer and the content of GA directly affected the solubility in water. In addition, the amount of the GA-PNIPAM (ii) P1 was higher than that of the GA-PNIPAM (i) P1; the difference was 0.82%, and the difference of the LCST was only 0.1 °C. This phenomenon may be caused by the ability of hydroxy or amino acids to form hydrogen bonds with water being different.

### 3.3. Assessment of Cell Viability

**Figure 6:**
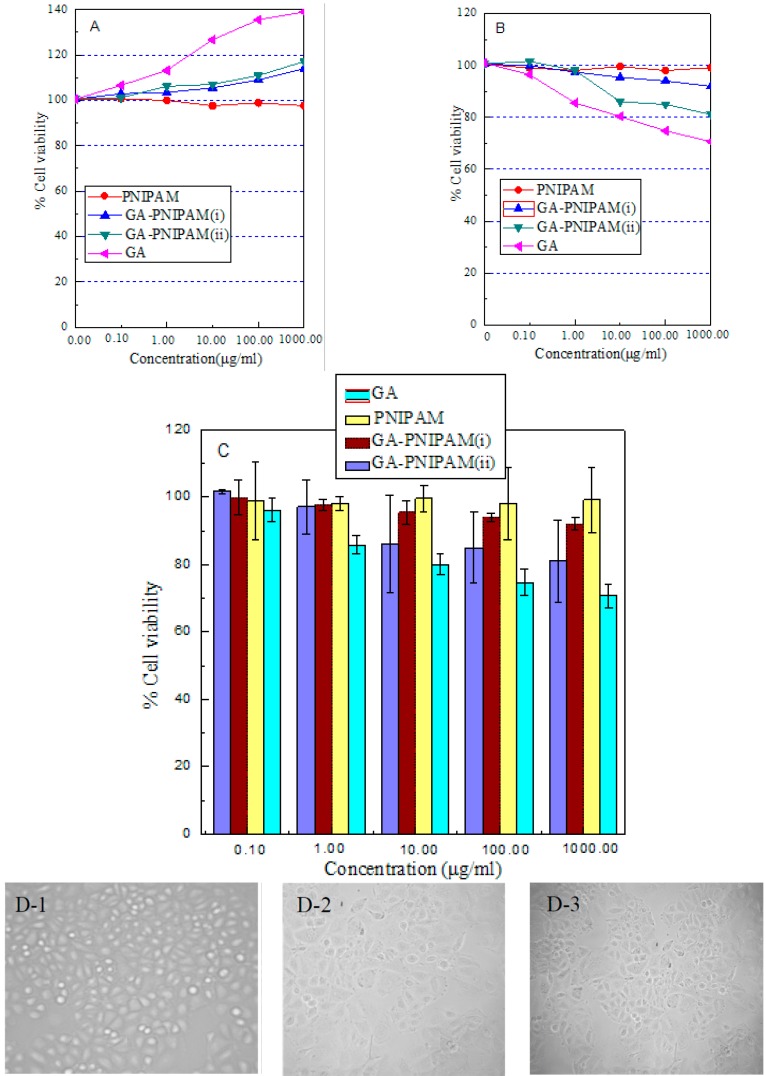
Cell viability of the (**A**) L-929 cells and (**B**) HepG2 cells incubated with the samples (PNIPAM homopolymer, GA-PNIPAM (i) and (ii); a range of sample concentrations from 0.1 to 1000 μg/mL) by MTT assay for 48 h; and the (**C**) HepG2 cells exposed to increasing concentrations of samples for different times; (**D**) Images of HepG2 cells with reference group (D-1), GA group (D-2) and GA-PNIPAM group (ii) (D-3) under microscope.

In our current study, by introducing the hydrophilic monomer GA into the PNIPAM chain, the LCST of the resultant polymers could be tuned to near body temperature for better biological application. So P4 of the GA-PNIPAM (i) and (ii) was used to evaluate the cytotoxicity. A cytotoxicity study on L-929 and HepG2 was conducted to investigate the biocompatibility of the PNIPAM homopolymer, GA, GA-PNIPAM (i) and (ii). The percent of cell viability was determined by comparison with cells that were not exposed to samples, which were used as the control group. No statistically significant cytotoxicity of the PNIPAM homopolymer was observed in cells, as shown in Figure 6A,B. Neither the P4 GA-PNIPAM polymer (i) nor (ii) was toxic in either cell line over a broad concentration range from 0.10 to 1000.00 μg/mL, and no clear difference between polymers was observed with regard to cytotoxicity to L-929. The viability of the HepG2 cells decreased to approximately 90% for GA-PNIPAM (i), whereas the value was only 80% for GA-PNIPAM (ii). It showed that GA-PNIPAM (ii) had suppressing activity for cancer cells; Figure 6C also shows evidence of this, which may be due to the amino groups present in GA-PNIPAM (ii). From Figure 6B,C), GA (reduction by sodium methylate) could effectively kill tumor cells, but the suppressing activity of GA-PNIPAM (ii) was weaker than that of GA. This might be due to the GA content of the polymer that was synthesized by ATRP being lower than that of the pure GA. Furthermore, at each concentration level of the dispersions of the GA and GA-PNIPAM (ii), cell survival decreased considerably as the time increased, as shown in Figure 6C, and cell activity was decreased along with the concentration. GA and GA-PNIPAM (ii) could change the growth state of HepG2 cells at the concentration of 1000.00 μg/mL after feeding 24 has shown in Figure 6D. HepG2 cells had a good condition and grew vigorously at the initial experiment (Figure 6D-1); when the GA and GA-PNIPAM (ii) were incubated in the cells, a large number of necrotic cells appeared, especially in the GA group. These indicated that GA-PNIPAM (ii) had the inhibition of tumor cell growth, and it could be a new smart material for antitumor effects and it may enhance the biocompatibility of GA for biomedicine.

## 4. Conclusions

Two end-functionalized PNIPAMs were synthesized via ATRP using different GA initiators which prepared through the amino acid at C-2 and the hydroxy at C-1. The GA-PNIPAM (i) and (ii) had a narrow dispersity and thermo-responsive property; their LCSTs were higher than that of the NIPAM homopolymer and these were attributed to the incorporation of the hydrophilic GA. The MTT assay showed that GA-PNIPAM (i) and (ii) had no toxicity for cells, and GA-PNIPAM (ii) had inhibitory activity for the tumor cells and no effect for normal cells, and thus it maybe have a potential application in antitumor therapy. 

## Figures and Tables

**Table 1:** Polymerization of GA-PNIPAM (i) and (ii).

Time (h)	GA-PNIPAM (i)			GA-PNIPAM (ii)		
	Conversion (%)	$M_{n}$_GPC_^a^	$M_{w}/M_{n}$_GPC_^a^	Conversion (%)	$M_{n}$_GPC_^a^	$M_{w}/M_{n}$_GPC_^a^
1	12.7	1710	1.17	11.6	1570	1.14
3	22.5	2590	1.14	20.1	2340	1.09
6	48.3	4190	1.18	49.4	4430	1.10
9	58.8	4880	1.16	61.5	5620	1.17
12	69.6	6100	1.14	69.3	6680	1.15

^a^ Determined by GPC using polystyrene standards.

**Table 2:** Characteristics of GA-PNIPAM (i) and (ii).

Sample	Time (h)	GA-PNIPAM (i)			GA-PNIPAM (ii)		
		$M_{n}$_GPC_^a^	GA (%)	LCST (°C)	$M_{n}$_GPC_^a^	GA (%)	LCST (°C)
P1	1	1710	19.19	43.6	1570	20.01	43.7
P2	3	2590	12.67	42.1	2340	13.42	41.8
P3	6	4190	7.83	38.8	4430	7.09	38.1
P4	9	4880	6.72	36.9	5620	5.59	36.6
P5	12	6100	5.37	34.7	6680	4.70	34.5

^a^ Determined by GPC using polystyrene standards.

## References

[1] Kazuyuki H., Takashi Y., Toshiyuki U. (1998). Ring-opening polymerization of new 1,6-anhydro-*β*-d-glucosamine derivatives. Carbohydr. Polym..

[2] Akiyama K., Kawazu K., Kobayashi A. (1995). A novel method for chemo-enzymatic synthesis of elicitor-active chitosan oligomers and partially N-deacetylated chitin oligomers using N-acylated chitotrioses as substrates in a lysozyme-catalyzed trans-glycosylation reaction system. Carbohydr. Res..

[3] Bertozzi C.R., Kiessling L.L. (2001). Chemical glycobiology. Science.

[4] Rudd P.M., Elliott T., Cresswell P., Wilson L.A., Dwek R.A. (2001). Glycosylation and the immune system. Science.

[5] Sears P., Wong C.H. (2001). Toward automated synthesis of oligosaccharides. Science.

[6] Friedman S.J., Skehan P. (1980). Membrane-active drugs potentiate the killing of tumor cells by d-glucosamine. Proc. Natl. Acad. Sci. USA.

[7] Molnar Z., Bekesi J.G. (1972). Effects of d-glucosamine, d-mannosamine, and 2-deoxy-d-glucose on the ultrastructure of ascites tumor cells in vitro. Cancer Res..

[8] Molnar Z., Bekesi J.G. (1972). Cytotoxic effects of d-glucosamine on the ultrastructures of normal and neoplastic tissues in vivo. Cancer Res..

[9] Reginster J.Y., Deroisy R. (2001). Glucosamine for osteoarthritis: Dawn of a new era. Lancet.

[10] Posakony J.J., Adrian R.F. (2013). Glucosamine and glucosamine-6-phosphate derivatives: Catalytic Cofactor Analogues for the glmS Ribozyme. J. Org. Chem..

[11] Li Y.Z., Rethwisch D.G. (2002). Scale-up of pseudo solid-phase enzymatic synthesis of alpha-methyl glucoside acrylate. Biotech. Bioeng..

[12] Nisao M.D., Pedatella S., Bektas S., Nucci A., Caputo R. (2012). d-Glucosamine in a chimeric prolinamide organocatalyst for direct asymmetric aldol addition. Carbohydr. Res..

[13] Wang X., Lin Y., Zeng Y., Sun X., Gong T., Zhang Z. (2013). Nano-lipoidal carriers of tretinoin with enhanced percutaneous absorption, photostability, biocompatibility and anti-psoriatic activity. Int. J. Pharm..

[14] Hong P.K., Gottardi D., Ndagijimana M., Betti M. (2014). Glycation and transglutaminase mediated glycosylation of fish gelatin peptides with glucosamine enhance bioactivity. Food Chem..

[15] Hrynets Y., Maurice N., Betti M. (2014). Food hydrocolloids, Transglutaminase-catalyzed glycosylation of natural actomyosin (NAM) using glucosamine as amine donor: Functionality and gel microstructure. Food Hydrocoll..

[16] Danac R., Ball L., Gurr S.J., Muller T., Fairbanks A. (2007). Carbohydrate chain terminators: Rational design of novel carbohydrate-based antifungal agents. J. ChemBioChem.

[17] Tan H.P., Ramirez C.M., Miljkovic N., Li H., Rubin J.P., Marra K.G. (2009). Thermosensitive injectable hyaluronic acid hydrogel for adipose tissue engineering. Biomaterials.

[18] Teng D.Y., Hou J.L., Zhang X.G., Wang X.Z., Li C.X. (2008). Glucosamine-carrying temperature- and pH-sensitive microgels: Preparation, characterization, and in vitro drug release studies. J. Coll. Interface Sci..

[19] Schild H.G. (1992). Poly(*N*-isopropylacrylamide)-experiment, theory and application. Polym. Sci..

[20] Gil E.S., Hudson S.M. (2004). Stimuli-reponsive polymers and their bioconjugates. Prog. Polym. Sci..

[21] Wang D., Liu T., Yin J., Liu S.Y. (2011). Stimuli-responsive fluorescent Poly(*N*-isopropylacrylamide) microgels labeled with phenylboronic acid moieties as multifunctional ratiometric probes for glucose and temperatures. Macromolecules.

[22] Gody G., Boullanger P., Ladaviere C., Charreyre M.T., Delair T. (2008). Biotin alpha-end-functionalized gradient glycopolymers synthesized by RAFT copolymerization. Macromol. Rapid Commun..

[23] Cui G.H., Li Y.H., Shi T.T., Gao Z.G., Qiu N.N., Satoh T., Kakuchi T., Duan Q. (2013). Synthesis and characterization on Eu(III) complexes of modified cellulose and poly(*N*-isopropylacrylamide). Carbohydr. Polym..

[24] Emmadi M., Kulkarni S.S. (2011). Rapid transformation of d-Mannose into orthogonally protected d-Glucosamine and d-Galactosamine thioglycosides. J. Org. Chem..

[25] Narain R., Armes S.P. (2002). Synthesis of low polydispersity, controlled-structure sugar methacrylate polymers under mild conditions without protecting group chemistry. Chem. Commun..

[26] Housni A., Cai H.J., Liu S.Y., Pun S.H., Narain R. (2007). Facile preparation of glyconanoparticles and their bioconjugation to streptavidin. Langmuir.

[27] Sun Y.S., Nitz M. (2012). syntheses of carbocyclic analogues of α-d-Glucosamine, α-d-Mannose, α-d-mannuronic acid, *β*-l-Idosamine, and *β*-l-Gulose. J. Org. Chem..

[28] Nakamatu K. (1982). Synthesis, luminescence quantum yields, and lifetimes of trischelated ruthenium (II) mixed-ligand complexes including 3,3′-Dimethyl-2,2′-bipyridyl. Bull. Chem. Soc. Jpn..

